# The effect of an acute bout of resistance exercise on carotid artery strain and strain rate

**DOI:** 10.14814/phy2.12959

**Published:** 2016-09-13

**Authors:** Jane M. Black, Eric J. Stöhr, Keeron Stone, Christopher J. A. Pugh, Mike Stembridge, Rob Shave, Joseph I. Esformes

**Affiliations:** ^1^ Cardiff School of Sport Cardiff Metropolitan University Cardiff United Kingdom; ^2^ Cardiff Centre for Exercise and Health Cardiff Metropolitan University Cardiff United Kingdom

**Keywords:** Arterial stiffness, circumferential strain, hemodynamics, strain rate

## Abstract

Arterial wall mechanics likely play an integral role in arterial responses to acute physiological stress. Therefore, this study aimed to determine the impact of low and moderate intensity double‐leg press exercise on common carotid artery (CCA) wall mechanics using 2D vascular strain imaging. Short‐axis CCA ultrasound images were collected in 15 healthy men (age: 21 ± 3 years; stature: 176.5 ± 6.2 cm; body mass; 80.6 ± 15.3 kg) before, during, and immediately after short‐duration isometric double‐leg press exercise at 30% and 60% of participants' one‐repetition maximum (1RM: 317 ± 72 kg). Images were analyzed for peak circumferential strain (PCS), peak systolic and diastolic strain rate (S‐SR and D‐SR), and arterial diameter. Heart rate (HR), systolic and diastolic blood pressure (SBP and DBP) were simultaneously assessed and arterial stiffness indices were calculated post hoc. A two‐way repeated measures ANOVA revealed that during isometric contraction, PCS and S‐SR decreased significantly (*P *<* *0.01) before increasing significantly above resting levels post exercise (*P *<* *0.05 and *P *<* *0.01, respectively). Conversely, D‐SR was unaltered throughout the protocol (*P *=* *0.25). No significant differences were observed between the 30% and 60% 1RM trials. Multiple regression analysis highlighted that HR, BP, and arterial diameter did not fully explain the total variance in PCS, S‐SR, and D‐SR. Acute double‐leg press exercise is therefore associated with similar transient changes in CCA wall mechanics at low and moderate intensities. CCA wall mechanics likely provide additional insight into localized intrinsic vascular wall properties beyond current measures of arterial stiffness.

## Introduction

Arterial responses to acute physiological stress are influenced by sympathetic neural control, hemodynamic conditions inside the vessel, and arterial wall mechanics. Arterial wall mechanics refer to the deformation and rate of deformation of an arterial wall in longitudinal, circumferential, and radial planes (Oishi et al. 2008; Kim et al. 2009; Yuda et al. 2011). Due to the progressive change in arterial structure from large arteries to small arterioles, there is currently no gold standard approach for the assessment of localized arterial stiffness, which describes the capacity of an artery to expand and contract in response to pressure changes (Bjällmark et al. 2010; Cecelja and Chowienczyk 2012). Furthermore, current techniques used to measure arterial stiffness such as pulse wave velocity do not permit the examination of localized arterial wall characteristics, and do not allow for the differences between systole and diastole to be examined. In contrast, novel two‐dimensional (2D) vascular strain imaging quantifies vascular tissue motion during systole and diastole by identifying markers (speckles) in the traditional gray‐scale ultrasound image and subsequently tracking these throughout the cardiac cycle (Bjällmark et al. 2010). Peak circumferential strain (deformation), peak systolic strain rate, and peak diastolic strain rate (the rate of deformation during systole and diastole) are measured directly from the motion of the arterial wall (Oishi et al. 2008; Kim et al. 2009; Yuda et al. 2011). Two‐dimensional vascular strain imaging has previously been validated in vitro in the longitudinal, radial and circumferential planes (Larsson et al. 2015). In vivo*,* common carotid artery (CCA) circumferential strain imaging has been shown to have the highest feasibility and reproducibility, and is significantly related to measures of arterial stiffness including *β* stiffness index, distensibility coefficient, and brachial‐ankle pulse wave velocity (Yuda et al. 2011). The application of this technique may therefore reveal valuable and novel insight into CCA wall mechanics at rest and during physiological stress in different populations. For example, this technique has previously been shown to differentiate arterial wall mechanics between young (<30 years) and older adults (>50 years) (Bjällmark et al. 2010). In older adults, degeneration of elastic fibers and compensatory increases in arterial wall collagen are known to occur (Oishi et al. 2008). In the study of Bjällmark et al. (2010), this was reflected in significant reductions in resting CCA peak circumferential strain (PCS), as well as peak systolic and diastolic strain rates (S‐SR and D‐SR, respectively) in the older adults compared to the younger adults. The authors suggest that a higher strain rate is beneficial as this may be indicative of a greater arterial elasticity (Bjällmark et al. 2010). It is therefore possible that the use of circumferential strain imaging to assess arterial wall mechanics might complement existing measures of arterial stiffness by providing a sensitive, noninvasive method to examine the localized intrinsic properties of the arterial wall between populations, at rest and during physiological stress.

During acute resistance exercise, arteries are exposed to numerous stimuli including increased blood flow (Green et al. 2005), shear stress (Gonzales et al. 2008), and blood pressure (MacDougall et al. 1985; Gotshall et al. 1999), as well as mechanical compression as a result of muscular contraction (MacDonald 2002). These responses have been shown to occur locally in the artery of the exercising limb (Gonzales et al. 2008, 2011) as well as remotely in arteries located in non‐exercising tissues (Thijssen et al. 2009; Totosy Zepetnek et al. 2015). CCA arterial stiffness is also known to increase following an acute bout of resistance exercise (Lefferts et al. 2014). Despite this, and the well‐known impact of acute resistance exercise on arterial hemodynamics, the effect of an acute bout of resistance exercise on CCA wall mechanics is not known. An understanding of how arterial wall mechanics change during an acute bout of resistance exercise, when blood pressure is significantly elevated, might provide insight into the mechanisms responsible for the increase in arterial stiffness previously reported (Lefferts et al. 2014). This is of particular importance in the CCA, as the brain is extremely susceptible to hemodynamic pulsatility (Hirata et al. 2006; Mitchell 2008) and a reduction in the ability to buffer elevations in both blood pressure and flow have been associated with an increased risk of stroke (Mattace‐Raso et al. 2006; Yang et al. 2012). Investigation of CCA wall mechanics during resistance exercise might also provide further insight into the specific mechanisms that underpin training‐induced vascular remodeling of the CCA, characterized by a decreased wall thickness (Spence et al. 2013) and increased diameter (Stebbings et al. 2013).

Based on the above considerations, the primary aim of this study was to examine changes in PCS, S‐SR, and D‐SR in the CCA during an acute bout of double‐leg press exercise. Consequential to the significant increase in heart rate (HR), blood pressure, and arterial diameter, it was hypothesized that PCS, S‐SR, and D‐SR would decrease significantly during the double‐leg press, before returning to baseline immediately post exercise. It was also hypothesized that more pronounced changes would occur during moderate‐ versus low‐intensity exercise. A secondary aim of the study was to investigate whether CCA PCS, S‐SR, and D‐SR at rest, during isometric contraction, and immediately post exercise are dependent on HR, blood pressure, and CCA diameter. It was hypothesized that HR, blood pressure, and CCA diameter would only partly explain PCS, S‐SR, and D‐SR and as such, these novel parameters could partially reflect acute alterations to the localized intrinsic properties of the CCA wall during physiological stress.

## Methods

### Participants

A total of 15 healthy recreationally active men (age: 21 ± 3 years; stature: 176.5 ± 6.2 cm; mass; 80.6 ± 15.3 kg; leg press 1 RM: 317 ± 72 kg) volunteered to participate and provided written informed consent prior to testing. All participants were nonsmokers, normotensive, had no previous history of cardiovascular, musculoskeletal, or metabolic disease, and were not taking any prescribed medication. The study protocol was approved by the Cardiff Metropolitan University School of Sport Research Ethics Committee, and adhered to the Declaration of Helsinki (2008).

### Experimental procedures

Participants reported to the laboratory on two separate occasions and were asked to refrain from strenuous exercise, alcohol, and caffeine intake for 24 h prior to each visit. During the first visit, the participants' 1 repetition maximum (1 RM) was determined for the double‐leg press exercise in accordance with guidelines set by the National Strength and Conditional Association (Baechle 1994), without the use of a Valsalva maneuver. During the second visit, participants' body mass and stature were recorded and a standardized warm‐up protocol was completed consisting of one set of 10 repetitions at both 10% 1 RM and 20% 1 RM with a 2‐min rest period between each set. Participants were then seated on the leg‐press machine where they rested for 5 min, while a cuff was attached to the middle phalanx of the middle finger of the right hand for continuous beat‐by‐beat measurement of BP (FinometerPro, FMS, Amsterdam, Netherlands), and HR was recorded via ECG (Vividq; GE Medical Systems Israel LTD). A single trained sonographer collected 2D gray‐scale images of the CCA short‐axis (1) before, (2) during isometric contraction, and (3) immediately (~12 sec) after double‐leg press exercise equal to 30% and 60% of 1 RM. At both exercise intensities, two repetitions were completed, each beginning with a dynamic leg extension. Subsequently, each participant was instructed to simultaneously lower the double‐leg press to a predetermined, standardized position (knee flexion angle of 90°), while exhaling to natural end‐expiration (functional residual capacity). The participant was then verbally instructed to hold this position (~5 sec) for image acquisition during isometric effort, before repeating the dynamic leg extension to complete the repetition. Simultaneous collection of all vascular and hemodynamic measurements ensured that variables were time aligned throughout the protocol. The order of intensity was randomized and counterbalanced throughout the experiment. Images were collected using a commercially available ultrasound system with a 12‐MHz linear array transducer (Vividq, GE Medical Systems Israel Ltd., Tirat Carmel, Israel) and all exercise was performed on a commercially available leg‐press machine (Linear Leg Press, Life Fitness (UK) ltd, Queen Adelaide, UK).

### Vascular ultrasonography and 2D strain imaging

**Figure 1:**
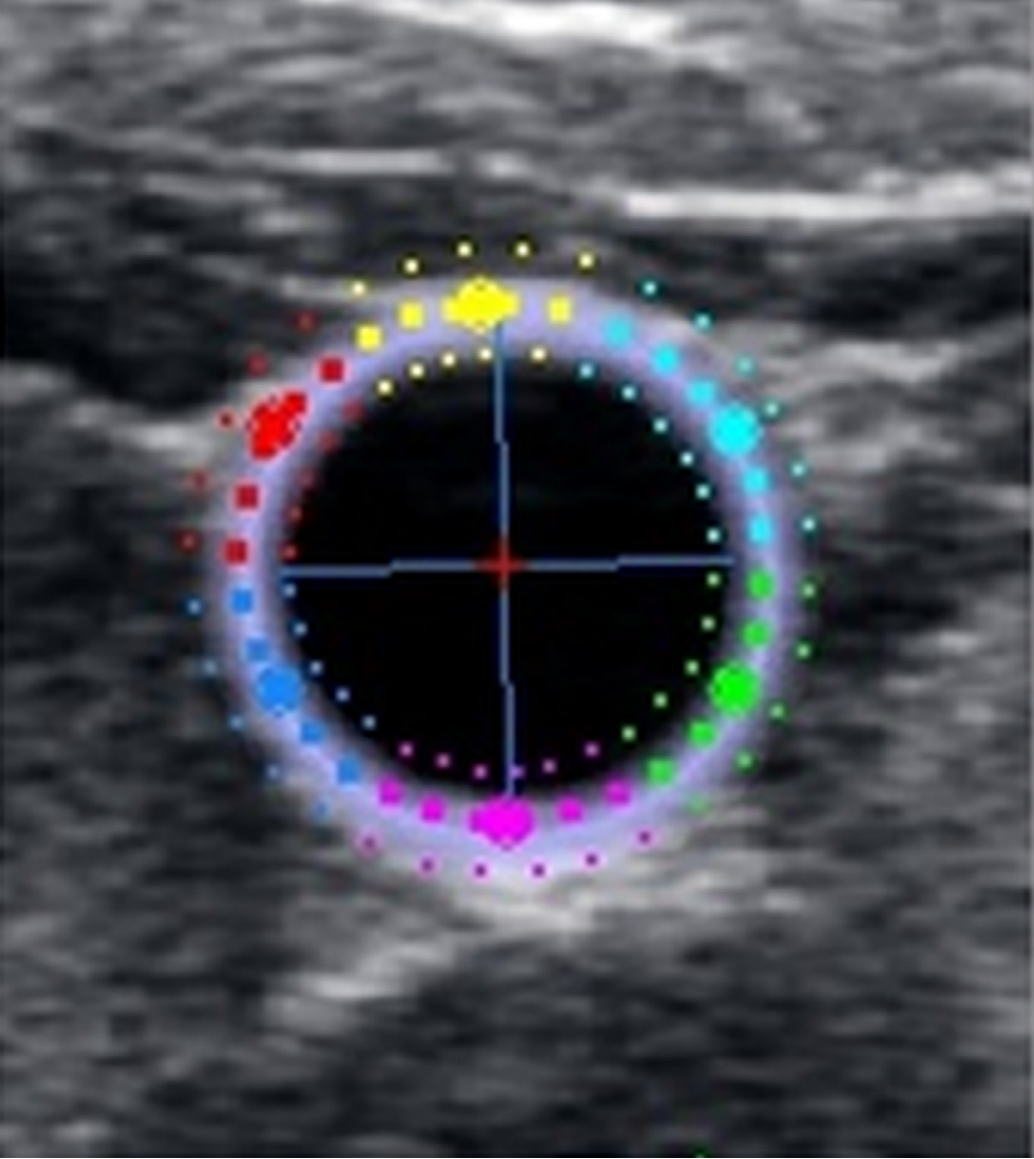
A region of interest placed over a cross‐sectional short‐axis image of the common carotid artery.

**Figure 2:**
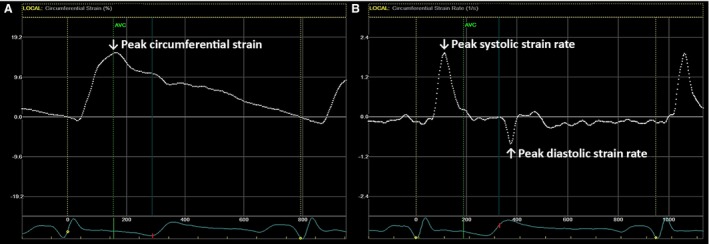
(A) Example of a circumferential strain curve produced following speckle tracking of the common carotid artery (CCA); and (B) example of a circumferential strain rate curve produced following speckle tracking of the CCA; Global measurements are represented by white, dotted lines; PCS, peak S‐SR, and peak D‐SR are labeled using white arrows.

Two‐dimensional short‐axis gray‐scale cine loops of the CCA were recorded 1–2 cm below the carotid bulb over a minimum of three consecutive cardiac cycles, and stored for subsequent offline analysis using dedicated 2D‐strain software (EchoPac Version 112, GE Vingmed Ultrasound, Horten Norway). Two‐dimensional strain software quantifies vascular tissue motion by automatically identifying speckles in the ultrasound image, which are subsequently tracked across the cardiac cycle (Bjällmark et al. 2010). PCS of the CCA (which reflects the circumferential deformation of the arterial wall from diastole to peak systole), peak S‐SR and D‐SR (which reflect the maximal rate of circumferential deformation during systole and diastole, respectively) were determined by manually placing a region of interest (ROI) over the cross‐sectional area of the CCA, and subsequently adjusting the ROI to ensure accurate alignment with the arterial wall. Within this ROI, movement of the speckles was tracked frame by frame throughout systole and diastole using a speckle tracking algorithm inherent to the software (Fig. 1), which resulted in the production of strain and strain rate curves (Fig. 2A and B, respectively). Adequate tracking of the CCA was objectively verified according to a quality assurance tool inherent to the software, and also visually confirmed by the operator, who manually adjusted the ROI, if necessary. All offline analyses were completed by a single operator. PCS, S‐SR, and D‐SR, were measured as an average over the circumference of the CCA (the entire ROI), providing “global” values for each of the variables. As previously defined (Oishi et al. 2008), PCS was identified as the greatest peak in the circumferential strain curve (Fig. 2A), peak S‐SR was identified as the first positive peak in the strain rate curve which occurred after the QRS complex, and peak D‐SR was determined as the first negative peak in the strain rate curve which occurred after the T‐wave of the ECG (Fig. 2B*)*. All 2D strain measurements were averaged for three consecutive beats. Peak systolic and peak diastolic CCA diameters (Diam_SYS_ and Diam_DIAS_, respectively) were defined as the maximum and minimum diameters during the cardiac cycle. Arterial diameter was measured manually using calipers from the leading edge of the intima–lumen interface of the anterior wall to the leading edge of the lumen–intima interface of the posterior wall of the short‐axis image (Oishi et al. 2008, 2011), thus ensuring that all CCA parameters (PCS, S‐SR, D‐SR, Diam_SYS_, and Diam_DIAS_) were measured from the same image.

To determine changes in stiffness of the CCA throughout the double‐leg press exercise, Peterson's elastic modulus (*E*_p_), *β*_1_ stiffness index, and *β*_2_ stiffness index were calculated as follows:

(1) $$E_{p}=\;\left(\text{SBP}-\text{DBP}\right)/\left(\left({\text{Diam}}_{\mathrm{SYS}}-{\text{Diam}}_{\mathrm{DIAS}}\right)/{\text{Diam}}_{\mathrm{DIAS}}\right)\;\;\text{in kpa}$$

(2) $$\beta_{1}=\;ln\left(\text{SBP}/\text{DBP}\right)/\left(\left({\text{Diam}}_{\mathrm{SYS}}-{\text{Diam}}_{\mathrm{DIAS}}\right)/{\text{Diam}}_{\mathrm{DIAS}}\right)\;\;\text{in}\;\;{\text{cm}}^{2}/\text{kpa}$$

(3) $$\beta_{2}=ln\left(\text{SBP}-\text{DBP}/\text{PCS}\right)$$

where SBP and DBP are systolic and diastolic blood pressures, respectively, and *ln* refers to the natural logarithm function. *E*_p_ and *β*_1_ are conventional measures of arterial stiffness and adjust changes in arterial diameter throughout the cardiac cycle for changes in distending pressure (Laurent et al. 2006). *β*_2_ incorporates measured peak circumferential strain and relates this to distending pulse pressure (Oishi et al. 2008, 2011). An increase in *E*_p_, *β*_1_, and *β*_2_ stiffness indices is indicative of a greater arterial stiffness compared with baseline in this particular anatomical region, at a given point in time.

### Statistical analysis

The reproducibility of PCS, S‐SR, and D‐SR was assessed prior to the experimental protocol and the intraobserver variability was determined by calculating coefficients of variation (CV). Normality of experimental data was examined and confirmed using the Shapiro–Wilk test. A two‐way repeated measures ANOVA was used to identify differences in all variables between the three phases of the movement (pre lift, during isometric contraction, post lift) and exercise intensity (30% and 60% of 1 RM), followed by paired samples *t* tests to identify differences. Standard multiple regression analysis was used to determine whether CCA PCS, S‐SR, and D‐SR at rest, during, and immediately post exercise were dependent on HR, SBP, DBP, Diam_SYS_, or Diam_DIAS_. For all statistical analysis, SPSS version 19.0 (Chicago, IL) was used and significance was accepted at 0.05. Data are presented as means ± SD.

## Results

### Heart rate, blood pressure, and common carotid arterial diameter

**Figure 3:**
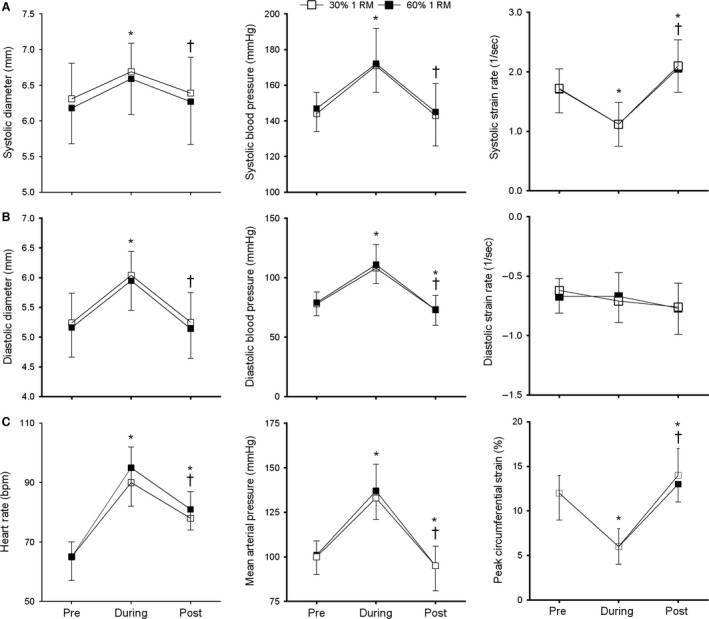
(A) Systolic parameters (Diam_SYS_, SBP, and S‐SR), (B) diastolic parameters (Diam_DIAS_, diastolic blood pressure, and D‐SR), and (C) heart rate (HR), mean arterial pressure (MAP), and PCS of the common carotid artery (CCA), at rest, during, and after double‐leg press exercise at 30% and 60% of 1RM (white and black squares, respectively). *P <* *0.05 versus pre; ^†^ *P *<* *0.05 versus during; values are means ±  SD.

During isometric contraction at both intensities, HR, SBP, DBP, Diam_SYS_, and Diam_DIAS_ increased significantly from baseline levels (all *P *<* *0.01, Fig. 3). Following exercise SBP, Diam_SYS_, and Diam_DIAS_ returned to baseline, whereas DBP dropped significantly below previous baseline levels (*P *<* *0.01). In contrast, HR decreased significantly after the double‐leg press (*P *<* *0.01), but remained significantly elevated following exercise in comparison with baseline (*P *<* *0.01). There were no statistically significant differences between the 30% and 60% 1 RM exercise intensities for any of the parameters examined (*P *>* *0.05).

### Arterial wall mechanics

During isometric contraction, PCS decreased from baseline at both 30% 1 RM and 60% 1 RM. The decrease in PCS was accompanied by a significant decrease in S‐SR at both 30% 1 RM and 60% 1 RM. Immediately post exercise, PCS and S‐SR increased significantly above baseline. In contrast, D‐SR remained unaltered throughout the experimental procedure (*P *=* *0.25, Fig. 3B). Again, no statistically significant differences were detected between the 30% and 60% 1 RM exercise trials for any of the arterial variables examined (*P *>* *0.05).

### Stiffness parameters

During isometric contraction *E*_p_, as well as *β*_1_ and *β*_2_ stiffness indices increased significantly from baseline (*P *<* *0.01, Table 1). Immediately post exercise, all three stiffness parameters returned to baseline. No statistically significant differences were identified between the 30% and 60% 1 RM exercise trials for any of the arterial stiffness variables examined (*P *>* *0.05).

**Table 1:** Stiffness parameters at rest, during isometric contraction, and immediately following double‐leg press exercise at 30% and 60% of 1 RM

Variable	30% 1 RM			60% 1 RM		
	Rest	During	Post	Rest	During	Post
***E***_**p**_ (kPa)	43.5 ± 11.7	88.4 ± 29.6^a^	43.8 ± 11.8^b^	47.6 ± 15.2	80.7 ± 24.3^a^	46.3 ± 14.9^a^
*β*_1_ stiffness index (mm^2^/kPa)	3.0 ± 0.7	4.9 ± 1.5^a^	3.2 ± 0.7^b^	3.3 ± 0.8	4.3 ± 1.1^a^	3.3 ± 0.8^b^
*β*_2_ stiffness index	1.7 ± 0.3	2.4 ± 0.5^a^	1.7 ± 0.3^b^	1.8 ± 0.3	2.3 ± 0.4^a^	1.7 ± 0.3^b^

*E*_p,_ Peterson's elastic modulus.

a *P *<* *0.05 versus pre.

b *P *<* *0.05 versus during; values are means ± SD.

### Determinants of arterial wall mechanics

Multiple regression analysis showed that collectively, HR, SBP, DBP, Diam_SYS_, and Diam_DIAS_ explained between 14‐81% of the total variance in PCS, S‐SR, and D‐SR at rest, 27–66% during isometric contraction and 39–53% immediately post exercise (Table 2). The regression analysis also highlighted significant individual predictor variables for PCS, S‐SR, and D‐SR at rest, during isometric contraction, and immediately post exercise, which are described below.

**Table 2:** Percentage of the total variance in arterial wall mechanics explained by the combination of HR, SBP, DBP, Diam_SYS_, and Diam_DIAS_ at rest, during isometric contraction, and immediately following double‐leg press exercise

Variable	Time	Total variance explained (%)	Effect size (*f* ^2^)	*P*‐value	Significant predictor variables
PCS	Pre	81	4.26	<0.01	Diam_SYS,_ and Diam_DIAS_
	During	66	1.94	<0.01	HR and SBP
	Post	53	1.13	<0.01	HR and DBP
S‐SR	Pre	65	1.86	<0.01	Diam_SYS,_ and Diam_DIAS_
	During	48	0.92	<0.05	—
	Post	39	0.64	<0.05	HR and DBP
D‐SR	Pre	14	0.16	0.62	—
	During	27	0.37	0.33	—
	Post	48	0.92	< 0.01	HR

Significant individual predictor variables for PCS, S‐SR, and D‐SR at rest, during, and immediately post exercise are discussed in the text.

PCS, peak circumferential strain; S‐SR, systolic strain rate; D‐SR: diastolic strain rate; Diam_SYS_, systolic CCA diameter; Diam_DIAS_, diastolic CCA diameter; SBP, systolic blood pressure; DBP, diastolic blood pressure; HR, heart rate.

#### Peak circumferential strain

At rest, Diam_SYS,_ and Diam_DIAS_ were identified as significant predictors of PCS (*β *= 1.78, *P < *0.01 and *β *= −1.97, *P < *0.01, respectively). In contrast, HR and SBP were identified as significant individual predictors for PCS during exercise (*β *= −0.72, *P < *0.05, and *β *= −0.38, *P < *0.05, respectively), whereas HR and DBP were identified as significant individual predictors of PCS immediately post exercise (*β *= 0.48, *P < *0.05, and *β *= −0.70, *P < *0.05, respectively).

#### Systolic strain rate

As with PCS, Diam_SYS,_ and Diam_DIAS_ were identified as significant individual predictors of S‐SR at rest (*β *= 1.18, *P < *0.01 and *β *= −1.08, *P < *0.01, respectively). No significant individual predictors were identified for S‐SR during isometric contraction, whereas HR and DBP were identified as significant individual predictors of S‐SR immediately post exercise (*β *= 0.60, *P < *0.01, and *β *= −0.58, *P < *0.05, respectively).

#### Diastolic strain rate

No significant individual predictors were identified for D‐SR at rest or during exercise, however, HR was identified as significant individual predictor of D‐SR immediately post exercise (*β *= −0.48, *P < *0.01).

### Reproducibility of measurements

The reproducibility of measurements of 2D strain imaging parameters was assessed in our laboratory. The CV for the intraobserver reliability of CCA PCS was 2.3%, which is considerably lower than the 3.9%, 5.8%, and 8.8% previously reported in the literature (Bjällmark et al. 2010; Yuda et al. 2011; Charwat‐Resl et al. 2015). The CV for the intraobserver reliability of S‐SR and D‐SR was 5.4% and 6.0%, respectively, which is in agreement with previously reported values (Bjällmark et al. 2010). The CV for the intraobserver reliability of *E*_p_ and *β*_1_ was 29% and 25%, respectively, which is higher than the 18% previously reported (Bjällmark et al. 2010). The CV for the intraobserver reliability of *β*_2_ was 18%. In accordance with previous research, the variability of the stiffness parameters (*E*_p_, *β*_1_, and *β*_2_) was considerably higher than that of PCS, S‐SR, and D‐SR (Bjällmark et al. 2010).

## Discussion

The aims of this study were to investigate changes in PCS, S‐SR, and D‐SR of the CCA in response to an acute bout of double‐leg press exercise and to examine whether PCS, S‐SR, and D‐SR at rest, during isometric contraction, and immediately post exercise were dependent on HR, blood pressure, and CCA diameter. The novel findings of this study were twofold: (1) an acute bout of double‐leg press exercise causes significant changes in CCA PCS and S‐SR but not D‐SR, during isometric contraction and immediately post exercise, and (2) HR, SBP, DBP, Diam_SYS_, and Diam_DIAS_ only partly explain the total variance in PCS, S‐SR, and D‐SR at rest, during isometric contraction, and immediately following an acute bout of double‐leg press exercise.

### Acute resistance exercise and common carotid arterial wall mechanics

Despite no change in D‐SR throughout the exercise protocol, PCS and S‐SR decreased significantly during isometric contraction; a finding which provides support for acute changes in systolic arterial wall mechanics in vessels located in non‐exercising tissues. As hypothesized, PCS and S‐SR (but not D‐SR) decreased significantly during isometric resistance exercise, and standard multiple regression revealed that this was partly explained by HR, blood pressure, and arterial diameter. Measurements obtained immediately after exercise showed that PCS significantly increased, exceeding baseline levels and this was accompanied by a significant increase in S‐SR, despite Diam_SYS_ and SBP returning to baseline. As previously suggested, it is possible that the increase in PCS and S‐SR observed immediately post exercise may indicate a greater arterial elasticity, as a result of acute changes to the intrinsic properties of the CCA wall (Bjällmark et al. 2010). The increase in S‐SR immediately post exercise may occur to buffer the elevated blood pressure associated with the onset of exercise, providing a smoother, more consistent flow (Kingwell 2002), and preventing damage to cerebral microvessels (O'Rourke and Safar 2005). The post exercise changes in both PCS and S‐SR shown in this study may also represent important stimuli for the chronic vascular remodeling observed following resistance training. Increased distension of the arterial wall has previously been shown to result in greater stretching of the load‐bearing lamellae, augmenting arterial wall stress (Laurent et al. 2001). Indeed, cyclic strain has been identified as a major determinant of the phenotype of vascular smooth muscle cells (VSMCs) in vitro (Laurent et al. 2001). Cyclic stretching has previously been shown to exert a greater influence on growth of the VSMCs than a static load, particularly in elastic arteries such as the CCA, where greater fluctuations in diameter occur throughout the cardiac cycle (Laurent et al. 2001). Despite there being no differences in post exercise arterial stiffness indices compared to baseline, the results of this study suggest that a bout of double‐leg press exercise causes acute increases in CCA wall deformation and the rate of deformation during systole, as evidenced by the significant increase in PCS and S‐SR immediately post exercise. Increased deformation of the CCA after an acute bout of resistance exercise may therefore represent the primary stimulus for the vascular remodeling associated with resistance training (Oishi et al. 2008; Spence et al. 2013). However, further research is needed to support this hypothesis.

The lack of differences in calculated arterial stiffness indices immediately post exercise helps to illustrate that 2D vascular strain imaging might be a more sensitive measure to accurately detect changes in localized arterial wall function following acute physiological stress. This technique may complement existing measures of arterial stiffness by providing additional insight into localized intrinsic vascular wall properties beyond current established measures.

### Determinants of arterial wall mechanics

As hypothesized, the multiple regression analysis revealed that HR, SBP, DBP, Diam_SYS_, and Diam_DIAS_ did not fully explain the total variance in PCS, S‐SR, and D‐SR at rest, during isometric contraction, or immediately post exercise. Resting PCS, for example, was largely explained by HR, blood pressure, and arterial diameter, whereas 61% of the total variance in S‐SR immediately post exercise was unexplained, despite both HR and DBP being identified as significant independent predictor variables. Similarly, between 52% and 86% of the total variance in D‐SR was not explained by HR, blood pressure, or arterial diameter. Therefore, although speculative, we suggest that some of the unexplained variance in arterial wall mechanics observed in this study might be attributed to acute intrinsic alterations of the vascular wall in response to physiological stress. It is thought that less than 10% of collagen fibers are engaged at rest, however, at higher pressures, such as during double‐leg press exercise, collagen fibers support wall tension, increasing arterial stiffness to prevent overstretching, and subsequent rupture of the arterial wall (Wagenseil and Mecham 2009). In support, an increase in arterial stiffness during isometric contraction (as evidenced by a rise in the stiffness indices calculated post hoc) was observed in this study. In contrast to previous research, this increase in arterial stiffness was transient and present only during isometric exercise (Lefferts et al. 2014). A shift toward stiffer collagen fibers, accompanied by an increase in HR and arterial diameter might therefore explain the reduction in PCS and S‐SR observed during isometric contraction. Immediately post exercise, the CCA becomes more distensible as arterial diameter and SBP return to baseline, and elastin rather than collagen, is primarily responsible for the transfer of stress through the CCA wall (Wagenseil and Mecham 2009). These changes, accompanied by an increase in pulse pressure and an elevation of HR, could account for the significant increases in PCS and S‐SR immediately post exercise.

In contrast to PCS and S‐SR, there was no significant change in CCA D‐SR throughout the exercise protocol, despite significant increases in diastolic arterial diameter and pressure during isometric contraction (Fig. 3B). This implies that CCA D‐SR is not influenced by resistance exercise‐induced changes in arterial diameter or pressure. In support, multiple regression analysis also highlighted that D‐SR was the parameter least influenced by HR, blood pressure, and arterial diameter both at rest and during isometric contraction. Previously, significant reductions in resting CCA D‐SR have been observed in older adults where degeneration of elastic fibers and compensatory increases in arterial wall collagen are known to occur (Bjällmark et al. 2010). Based on the results of this study, we therefore propose that D‐SR might be an important parameter to accurately reflect changes in the localized intrinsic properties of the arterial wall, independent of heart rate, blood pressure, and arterial diameter. In the future, examination of CCA D‐SR in elderly and clinical populations where changes in the composition of arterial wall collagen and elastin have occurred could provide support for this hypothesis. Additionally, further research is needed to determine the true independent individual influence of other physiological variables such as HR, stroke volume (SV), arterial diameter, blood pressure, pulse pressure, and mean arterial pressure on acute changes in arterial wall mechanics. Identification of the most influential physiological variables may allow for CCA PCS, S‐SR, and D‐SR to be normalized appropriately to the loading stimuli. In this study, no single physiological variable was identified as a significant predictor for CCA PCS, S‐SR, and D‐SR, and therefore normalization of these variables was not possible.

### Acute resistance exercise and arterial hemodynamics

As expected, and in accordance with previous research, a significant rise in HR, SBP, and DBP was observed during double‐leg press exercise (MacDougall et al. 1985; Gotshall et al. 1999; Mayo and Kravitz 1999). Immediately post exercise, SBP returned to baseline, whereas a significant drop in DBP below baseline levels was observed. The decrease in DBP might be attributed to a reduction in both the force of muscle contraction and intramuscular pressure on cessation of exercise. In contrast to previous research and our initial hypothesis, acute changes in arterial hemodynamics did not differ between exercise intensities (Fleck and Dean 1985; Wiecek et al. 1990; King et al. 2000). This may, however, be explained by the small number of repetitions, as previous research has highlighted that a single repetition at 100% 1 RM elicits less hemodynamic changes than a higher number of repetitions at a lower intensity (Wilborn et al. 2004).

### Acute resistance exercise and common carotid arterial diameter

Previous research investigating the influence of acute resistance exercise on CCA diameter have only reported values at rest and immediately post exercise (DeVan et al. 2005; Lefferts et al. 2014). Results of this study indicate that, irrespective of exercise intensity (30% and 60% 1 RM), both Diam_SYS_ and Diam_DIAS_ increased significantly during isometric resistance exercise, before returning to baseline immediately post exercise. CCA smooth muscle is known to be innervated by sympathetic efferents (Studinger et al. 2003) and significant increases in vasoconstrictor sympathetic nerve activity are known to occur during isometric exercise, even at low intensities (Fisher et al. 2006). In contrast to this, Diam_SYS_ and Diam_DIAS_ increased during isometric exercise in our trial, suggesting that distending forces supersede smooth muscle contraction (Studinger et al. 2003). This increase in CCA diameter during isometric resistance exercise is unlikely to have occurred as a result of an increase in SV, as previous research has consistently reported that SV remains unchanged or decreases during an acute bout of resistance exercise (Miles et al. 1987; Lentini et al. 1993; Elstad et al. 2009). While not measured during this study, published data from our laboratory has previously reported a decrease in SV during double‐leg press exercise, using a similar protocol (Stöhr et al. 2015), attributed to a combination of decreased preload and increased afterload (Lentini et al. 1993). The transient increase in CCA diameter observed in this study is likely related to the significant increase in blood pressure which has consistently been shown to occur during resistance exercise (MacDougall et al. 1985; Gotshall et al. 1999). In support, a significant relationship between changes in CCA mean diameter and pressure has previously been shown during strenuous dynamic exercise (Studinger et al. 2003).

### Limitations

The lack of data available on the acute cardiovascular responses to resistance exercise has previously been attributed to difficulties associated with accurate determination of vascular assessment during resistance exercise (Fleck 2003). To overcome this issue, images were collected during a brief isometric hold and while this lacks ecological validity, it does allow accurate data to be collected. Stiffness indices were calculated to relate the changes in arterial pressure and diameter, however, as blood pressure and arterial lumen diameter were measured in the brachial and carotid arteries, respectively, this could have resulted in an overestimation of arterial stiffness and must therefore be recognized as a limitation. We were unable to measure SV during this study and are therefore unable to draw firm conclusions about the interaction between cardiac and vascular responses to an acute bout of resistance exercise. In the future, simultaneous measurements of cardiac and vascular responses would be informative in developing a greater understanding of the acute cardiovascular responses to resistance exercise. Vascular responses to an acute bout of resistance exercise should also be measured in other arteries, both central and peripheral, to understand how responses differ in elastic and muscular arteries. Acute vascular responses during high‐intensity resistance exercise and the influence of training status should also be considered. Despite these limitations, 2D vascular strain imaging is a simple technique, which can provide additional insight into the mechanical behavior of the arterial wall, allowing for differences between systole and diastole to be examined, and regional comparisons to be made between arteries. While this technique is not likely to replace established and validated measures of arterial stiffness, it could complement existing measures and provide further insight into localized arterial wall function throughout the cardiac cycle, both within and between different populations.

## Conclusion

Using novel 2D vascular strain imaging, this study has shown that acute changes in systolic (PCS and S‐SR), but not diastolic (D‐SR) arterial wall mechanics occur in the CCA during and immediately post an acute bout of double‐leg press exercise, at both low and moderate intensities. The systolic responses may indicate greater elasticity of the CCA immediately post exercise, or reflect a protective mechanism to buffer the elevated blood pressure associated with the onset of resistance exercise, preventing damage to cerebral microvessels. In contrast, CCA D‐SR is not influenced by significant changes in arterial diameter or pressure during isometric resistance exercise and may therefore be an important parameter to accurately reflect changes in localized intrinsic vascular wall properties, although further research is still required.

## Conflicts of Interest

There is no funding to be declared in relation to this study and the authors have no conflict of interest.
